# Value of Combination Therapy With Antiepileptic Drugs (AEDs) for Migraine to Prevent Ischemic Stroke in Young Women

**DOI:** 10.7759/cureus.26972

**Published:** 2022-07-18

**Authors:** Faropoulos Konstantinos, Vasiliki Tsolaki, Vasiliki E Georgakopoulou, Aikaterini Aravantinou, George Fotakopoulos

**Affiliations:** 1 Neurosurgery, Nicosia General Hospital, Nicosia, CYP; 2 Pulmonary and Critical Care Medicine, University Hospital of Larissa, Larissa, GRC; 3 Pulmonology, Laiko General Hospital, Athens, GRC; 4 Pulmonology, Sismanogleio Hospital, Marousi, GRC; 5 Internal Medicine, Laiko General Hospital, Athens, GRC; 6 Neurosurgery, General University Hospital of Larissa, Larissa, GRC

**Keywords:** sumatriptan therapy, levacetam, antiepileptic agent, ischemic stroke, : migraine

## Abstract

The aim of this study was to systematically assess the efficacy of a combination of levetiracetam and sumatriptan as a preventive treatment for migraine and stroke in young women.

This was a retrospective study with 342 female patients, who experienced migraines. All patients were divided into three groups: Group A (124 patients (36.2%) with triptan (sumatriptan) monotherapy), Group B (111 patients (32.4%) with a combined triptan and antiepileptic drug (AED) therapy with levetiracetam (LEV)), and Group C (107 (31.2%) patients with placebo treatment), in a 1.4:1.1:1 ratio respectively.

Significant differences were found in Group B when compared with Groups A and C with respect to the use of combination hormonal contraception, smoking, a family history of migraine, and seizures.

The results of this study suggested that combination treatment with levetiracetam and sumatriptan was more effective in preventing migraines and reducing the risk of stroke in young women than sumatriptan monotherapy.

## Introduction

Migraine and ischemic stroke are two of very widespread entities, affecting females more commonly compared with male population [1]. Several studies have reported on the association between migraine and the relative risk of stroke in young women compared to the general population [2]. In contrast, the Women’s Health Study did not describe any relation between ischemic stroke and undifferentiated migraine, but in the subgroup that presented with active migraine with aura (MWA), the risk of ischemic stroke was found to be about twice as high as those with no migraine [3]. However, over the next years, the aura-ischemic stroke connection has been further confirmed in multiple case-control and prospective cohort studies, in migraine patients [4].

According to International Classification of Headache Disorders, 3rd edition, the migrainous infarction has been defined as the ischemic stroke event that had a neuroimaging correlate and occurred during a typical attack of MWA with at least one aura symptom lasting > 60 minutes without other apparent cause [5]. Studies suggested that the incidence of migraine-related stroke was between 1.4 and 1.7 per 100,000 person-years [6], representing 0.5 to 1.5% of all ischemic strokes [7], but up to 14% of them were diagnosed in young individuals (mean 36.7 years, range 24-50) and in those experiencing first ischemic stroke occurrence [8].

Triptans, are drugs that act as agonists for serotonin 5-HT1B and 5-HT1D receptors at blood vessels and nerve endings in the brain, and have been shown to be effective in acute treatment of migraine [9]. The possible mechanisms of their action are the vasospasm of cranial blood vessels, the reduction of neurogenic inflammation around blood vessels, and the neuropeptide release [10]. Sumatriptan has been approved by the Food and Drug Administration (FDA), at a dose of 50 mg, as acute migraine treatment medication [9]. Conflicting data about the use of triptans and the related risk of stroke have been published [11]. According to many authors, dysfunction of the endothelium, which may be a cause or a consequence of migraine, hypercoagulability, and inflammation were the potential mechanisms between migraine and ischemia at the microvascular level [12]. For this reason, triptans in MWA should be prescribed after aura subsides and headache begins, to avoid medication-related vasoconstriction during aura [10].

Cortical spreading depression (CSD) is thought to be critical in the pathophysiology of migraine representing an intense depolarization that underlies migraine aura, and which also occurs in peri-infarct tissue as an overlapping mechanism between migraine and stroke [13]. Although CSD does not cause injury in the healthy brain tissue, recurrent peri-infarct CSDs and depolarizations (PIDs) deteriorate the metabolic process in ischemic tissue and promote infarct growth during hyperacute stroke in both experimental animals and humans [13]. The general role of antiepileptic drugs in migraine prevention lies in their potential capacity to block CSD and prevents secondary injury [14].

Taking all these into account, the current study aims was to evaluate the efficacy of combination levetiracetam and sumatriptan as a preventive treatment for migraine and stroke to the young women. Moreover, the further analysis provides information about the risk factors for stroke in young women with migraine.

## Materials and methods

In this retrospective cohort study, we analyzed 342 out of 1042 female patients who were newly diagnosed with migraines in the tertiary teaching hospital, University Hospital of Larissa, Larissa, Greece, based on anonymized hospital records. The Institutional Review Board (IRB) of the Nicosia General Hospital, Nicosia, Cyprus, approved the study (IRB Number: 4416/12-01-2015, finalized by the 5th General Assembly on January 19, 2015).

A total of 342 eligible subjects were divided into three groups, namely: Group A (Tr; 124 patients (36.2%) with triptan (sumatriptan) monotherapy), Group B (TrA; 111 patients (32.4%) with a combined triptan and antiepileptic drug (AED) therapy with levetiracetam (LEV)), and Group C (Pl; 107 (31.2%) patients with placebo treatment), in a 1.4:1.1:1 ratio respectively. They had undergone treatment between January 2015 and February 2019 (four years). All patients had received therapy for 12 months and were evaluated with electroencephalography (EEG), magnetic resonance imaging (MRI), and a standard neurological examination. Participants with migraines provided information on their headache characteristics including the history of migraine, headache frequency (attacks per month), and headache intensity in the past month using the visual analog scale (VAS) with anchors at 0 and 10 where 0 = no pain, 10 = worst pain possible, VASpro (baseline), and VASpost (after 12 months).

Included patients fulfilled the International Classification of Headache Disorders-III criteria for migraine and had at least one migraine attack per month, which was identified by the attending neurologist/physician. Exclusion criteria included non-migraine headaches according to the International Classification of Headache Disorders-III criteria, a history of a neck injury, claustrophobia, or severe depression (i.e., Depression Anxiety Stress Scales-21, score > 21).

The patient’s outcome was evaluated with the difference between pre-therapy (baseline) VAS and post-therapy VAS (DVAS) and the difference between pre-therapy (baseline) migraine frequency and post-therapy migraine frequency (DFreq ). Negatives values or failure to improve on treatment were taken as 0.

Levetiracetam was initiated in participants at 250 mg/day for the first week and increased to a total dose of 500 mg/day (250 mg, twice daily) after one week. All patients were treated with sumatriptan at a dose of 50 mg. After 12 months of treatment, headache frequency and intensity were recorded and compared with the baseline values (from the first day of initiation).

The main MRI findings were the observation of brain infarcts, radiographically defined as T2 hyperintensity and T1 hypointensity lesions. Lesions were identified as small or multiple in a single MRI image in stroke patients.

## Results

In this study, 342 female patients were included. The baseline characteristics of the study participants are shown in Table 1. It is worth mentioning that the mean age was 36 years with a standard deviation of ±8.0.

**Table 1:** Baseline characteristics of patients Data are presented as mean ±SD, otherwise is indicated. P-value for the difference between groups was assessed for Nominal data using the Fisher’s exact test and for Continuous data with the Mann-Whitney U test as appropriate. Tr: triptans therapy; TrA: combined triptans and antiepileptic drugs therapy; MRI: magnetic resonance imaging; NSAIDs: nonsteroidal anti-inflammatory drugs;

Parameters	All patients, n= 342 (100%)	Group A: Tr, n= 124 (36.2%)	Group B: TrA, n= 111 (32.4%)	Group C: Pl, n=107(31.2%)	P-value
Age, years	36.7±8.0	36.0±8.1	37.0±8.1	37.2±7.8	0.533
Combined hormonal contraception use, n(%)	49 (14.3)	26 (7.6)	11 (3.2)	12 (3.5)	0.029
Elevated cholesterol, n(%)	16 (4.6)	4 (1.1)	4 (1.1)	8 (2.2)	0.252
Smoke, n(%)	68 (19.8)	35 (10.2)	19 (5.5)	14 (4.0)	0.011
Diabete, n(%)	21 (6.1)	7 (2.0)	8 (2.3)	6 (1.7)	0.850
Family history of migraine, n(%)	31 (9.0)	19 (5.5)	7 (2.0)	5 (1.4)	0.009
NSAIDs consummation, n(%)	76 (22.2)	29 (8.4)	26 (7.6)	21 (6.1)	0.738
Venous thromboembolism, n(%)	10 (2.9)	3 (0.8)	5 (1.4)	2 (0.5)	0.471
Hypertension, n(%)	29 (8.4)	10 (2.9)	9 (2.6)	10 (2.9)	0.927
Pregnancy, n(%)	4 (1.1)	2 (0.5)	1 (0.2)	1 (0.2)	0.847
Preeclampsia, n(%)	5 (1.4)	5 (1.4)	0 (0)	0 (0)	0.012
Atrial fibrillation (foramen ovale), n(%)	1 (0.2)	0 (0)	1 (0.2)	0 (0)	0.414
MRI brain lesions, n(%)	10 (2.9)	5 (1.4)	4 (1.1)	1 (0.2)	0.321
Antifibrolytic therapy, n(%)	10 (2.9)	3 (0.8)	5 (1.4)	2 (0.5)	0.471
Seizures, n(%)	9 (2.6)	7 (2.0)	1 (0.2)	1 (0.2)	0.032

Statistically significant differences were found between the two groups with respect to combination hormonal contraception use (p = 0.029), smoking (p = 0.011), a family history of migraine (p = 0.009), pre-eclampsia (p = 0.012), and seizures (p = 0.032) (Table 1).

Outcomes

Clinical outcomes are shown in Table 2.

**Table 2:** Headache of migraine patients before and after treatment between groups Data are presented as mean ±SD, otherwise is indicated. P-value for the difference between groups was assessed for Nominal data using the Fisher’s exact test and for Continuous data with the Mann-Whitney U test as appropriate. Tr: triptans therapy;TrA: combined triptans and antiepileptic drugs therapy; Freq(pro): pro-therapy (baseline) migraine frequency; Freq(post): post-therapy migraine frequency; VASpro: pro-therapy (baseline) visual analog scale; VASpost: post-therapy visual analog scale

Parameters	All patients, n= 342 (100%)		Group A: Tr, n= 124 (36.2%)	Group B: TrA, n=111 (32.4%)	Group C: Pl, n=107(31.2%)	P-value
Headache of migraine						
1. Frequency, times per month						
-Freq(pro)	5.3±1.9		5.4±2.0	5.3±1.9	5.1±1.8	0.597
-Freq(post)	4.3±2.0		5.1±2.0	3.1±1.9	4.6±1.5	0.000
2.Intensity						
-VASpro	6.7±1.2		6.7±1.2	6.8±1.2	6.7±1.1	0.725
-VASpost	4.9±2.1		6.1±1.3	2.4±1.1	6.1±1.2	0.000

The frequency and intensity of headaches were statistically significantly different between groups (p < 0.05). DVAS and DFreq were significantly different as well (p = 0.039 and p = 0.002, respectively) (Table 3).

**Table 3:** Outcomes of patients Data are presented as mean ±SD, otherwise is indicated. P-value for the difference between groups was assessed for Nominal data using the Fisher’s exact test and for Continuous data with the Mann-Whitney U test as appropriate. DVAS: difference between pre-therapy (baseline) visual analog scale and post-therapy visual analog scale; DFreq: difference between pre-therapy (baseline) migraine frequency and post-therapy migraine frequency

Parameters	All patients, n= 342 (100%)	Stroke, n = 13 (3.8%)	No stroke, n=329 (96.1%)	P-value
Migraine characteristics				
-DVAS	1.85±2.1	0.61±1.1	1.9±2.1	0.039
-DFreq	1.04±1.5	0.0±0.0	1.08±1.5	0.002

This means that the frequency of headaches between groups was different (in group B with combined therapy, it was less as compared with the others groups). Univariate analysis indicated that combined hormonal contraception use, a family history of migraine, venous thromboembolism, pregnancy and pre-eclampsia, and antifibrinolytic therapy were associated with stroke (Table 4).

**Table 4:** Univariate analysis for stroke Data are presented as mean ±SD, otherwise is indicated. P-value for the difference between groups was assessed for Nominal data using the Fisher’s exact test and for Continuous data with the Mann-Whitney U test as appropriate. Tr: triptans therapy; TrA: combined triptans and antiepileptic drugs therapy; MRI: magnetic resonance imaging; NSAIDs: nonsteroidal anti-inflammatory drugs

Parameters	Stroke, n=13 (3.8%)	No stroke, n=329 (96.1%)	Correlation P-value
Groups			
-Group A: Tr, n (%)	8(3.4)	116(49.3)	0.091
-Group B: TrA, n(%)	4(1.7)	107(45.5)	
-Group C: Pl, n(%)	1	106	
Age, years	34.15±7.9	36.84±8.0	0.260
Combined hormonal contraception use, n(%)	5 (2.1)	44 (13.6)	0.011
Elevated cholesterol, n(%)	0 (0)	16 (3.4)	0.415
Smoke, n(%)	1 (0.4)	67 (22.5)	0.262
Diabetes, n(%)	0 (0)	21 (6.3)	0.347
Family history of migraine, n(%)	4 (1.7)	27 (9.3)	0.005
NSAIDs consummation, n(%)	2 (0.8)	74 (22.5)	0.545
Venous thromboembolism, n(%)	5 (2.1)	5 (1.2)	0.000
Hypertension, n(%)	1 (0.4)	28 (7.2)	0.917
Pregnancy, n(%)	3 (1.2)	1 (0)	0.000
Preeclampsia, n(%)	2 (0.8)	3 (1.2)	0.000
Atrial fibrillation (foramen ovale), n(%)	0 (0)	1 (0.4)	0.842
MRI brain lesions, n(%)	0 (0)	10 (3.8)	0.523
Antifibrinolytic therapy, n(%)	5 (2.1)	5 (1.2)	0.000
Seizures, n(%)	0 (0)	9 (1.7)	0.546

**Figure 1:**
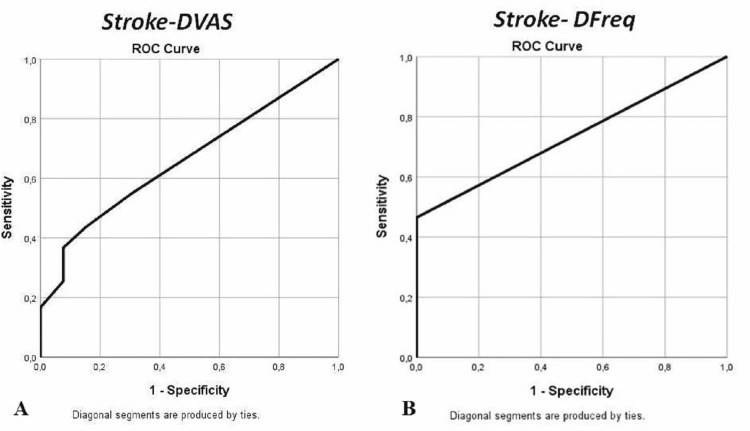
Receiver operating characteristic (ROC) analysis A) Receiver operating characteristic (ROC) analysis revealed that stroke and DVAS (difference between pre-therapy (baseline) visual analog scale and post-therapy visual analog scale) wasn’t an accurate measure to identify stroke with an area under curve standard error AUC(SE) of 0.660(.062), p = 0.051; B) receiver operating characteristic (ROC) analysis revealed that stroke and DFreq (difference between pre-therapy (baseline) migraine frequency and post-therapy migraine frequency) was one of the most accurate measures to identify stroke with an area under curve standard error AUC(SE) of 0.733(.047), p = 0.047

According to receiver operating characteristic analysis, DVAS wasn’t predictive of stroke with an area under the curve standard error (AUC(SE)) of 0.660(.062), p = 0.051 (Figure 1A). DFreq was one of the most accurate measures for identifying stroke with an AUC(SE) of 0.733(.047), p = 0.047, whereas a DFreq > 2.5 was presented with the best performance (80% sensitivity, 100% specificity) (Figure 1B).

Multivariate analysis indicated that a family history of migraine, venous thromboembolism, pregnancy/pre-eclampsia, and antifibrinolytic therapy were independent risk factors for stroke in young women with migraines (Table 5).

**Table 5:** Independent risk factors of stroke of patients with a migraine after multivariable analysis P=value for the difference between parameters was assessed for Nominal data using Fisher’s exact test and for Continuous data with the Mann-Whitney U test as appropriate. CI: conﬁdence interval; mMT: mini Mental test; DVAS: difference between pre-therapy (baseline) visual analog scale and post-therapy visual analog scale; DFreq: difference between pre-therapy (baseline) migraine frequency and post-therapy migraine frequency

Name			OR		CI(95%) lower-upper		P-value		
Combined hormonal contraception use			0.025		-0.020-0.069		0.271		
Family history of migraine			0.072		0.019-0.126		0.009		
Venous thromboembolism			0.276		0.126-0.426		0.000		
Pregnancy			0.728		0.584-0.871		0.000		
Preeclampsia			0.377		0.249-0.506		0.000		
Antifibrolytic therapy			0.264		0.113-0.414		0.001		
Statistical ﬁndings for ROC									
Parameters		Area		Standard Error		CI(95%) lower-upper		P-value	
Stroke-DVAS	0.660				0.062	0.538-0.782		0.051	
Stroke-DFreq	0.733				0.047	0.641-0.824		0.047	

## Discussion

The main findings of the present, large, retrospective cohort study, which included young women with migraines who were randomized to receive as a preventive treatment either sumatriptan (Group A), sumatriptan plus levetiracetam (Group B), or a placebo therapy (Group C), were the following: (1) There was an improvement in the frequency and intensity of headache in Group B as compared with Groups A and C (p < 0.05); (2) DFreq was one of the most accurate measures for identifying stroke with an AUC(SE) of 0.765(.045), p = 0.002, whereas a DFreq > 2.5 indicated the best performance (80% sensitivity, 100% specificity); (3) Significant risks for stroke in young women with migraines were found to be a family history of migraine (p = 0.023), venous thromboembolism (p < 0.05), pregnancy/pre-eclampsia (p < 0.05), and antifibrinolytic therapy (p < 0.05).

According to the Headache Treatment Guidelines, patients who have high frequent episodes of migraines are candidates for receiving preventive drugs [15], even though only 30% actually use them [16]. The negative effects of this condition on individuals, their families, and society are multiple [16]. Moreover, these patients are at a high risk of developing chronic daily headaches [17]. In agreement with the 2000 US Headache Consortium, the best preventive treatment should decrease attack frequency, intensity, and duration, leading to a better response to acute treatment, a decline in disability, and prevention of medication overuse headache (MOC) [18]. Our data are important because the frequency and intensity of headaches were decreased more in Group B, which used the combination treatment, than in Groups A and C.

As mentioned previously, another significant observation in our study was that DFreq was found to be another precise attribute that identified risk for stroke, and only patients with combined migraine therapy had an improvement in migraine frequency that was greater than 2.5 times per month (DFreq > 2.5), showing a very low probability of developing a stroke. Thus, our study demonstrated that the combination therapy with levetiracetam and sumatriptan was more effective, not only as a preventive cure for migraines but also as a prophylactic treatment for stroke.

Identification of the independent risk factors in young women with migraines to develop stroke is of great importance because it can lead to major preventive interventions. In addition, whereas traditional vascular risk factors are for both genders, our study showed that there are specific risk factors for stroke that occur only in women. Endogenous hormones play a major role at the age of menarche and particularly age ≥ 17 increases the risk for a cerebral accident [19]. Combined oral contraceptive drugs and pregnancy/pre-eclampsia have also been associated with an increased risk for stroke [20]. Women who take contraception drugs and have migraines with aura have an amplified potential risk [21]. The independent risk factors for stroke in the specific population that was included in our study were a family history of migraine, venous thromboembolism, pregnancy/pre-eclampsia, and antifibrinolytic therapy (Table 5).

As the incidence of migraine-related stroke is between 1.4 and 1.7 per 100,000 person-years [6], headache pain intensity is as significant as the frequency when evaluating the clinical response and impact on patient headache-related disability after migraine preventive treatment [22]. But in our study, according to receiver operating characteristic analysis, DVAS wasn’t predictive of stroke and migraine preventive treatment.

There were certain limitations in the present study. First, it was based in one, small center, and was not a multicenter clinical trial. In this respect, the efficacy of the combination treatment as a preventive treatment for migraine and stroke cannot be generalized. However, this study could be the basis for future, larger, clinical trials. Second, we did not eliminate all the confounding factors, possibly due to the small sample size, and this may have affected the outcome.

## Conclusions

Combination therapy with levetiracetam and sumatriptan was shown to reduce the frequency and intensity of migraine and prevent stroke in young women. However, this promising outcome, which is of significance for physicians when considering preventive treatments, needs further exploration with larger, multicenter studies. Future studies including large, multicenter, double-blind, randomized control trials are important to systematically assess the efficacy of a combination of levetiracetam and sumatriptan as preventive treatment for migraine and stroke in young women.
